# p42.3 gene expression in gastric cancer cell and its protein regulatory network analysis

**DOI:** 10.1186/1742-4682-9-53

**Published:** 2012-12-11

**Authors:** Jianhua Zhang, Chunlei Lu, Zhigang Shang, Rui Xing, Li Shi, Youyong Lv

**Affiliations:** 1 Department of Biomedical Engineering, School of Electrical Engineering, Zhengzhou University, Zhengzhou, Henan Province, 450001, China; 2 Department of Gastrointestinal Surgery, PLA No.101Hospital, No.101, North Xingyuan Road, Wuxi, Jiangsu Province, 214044, China; 3 Laboratory of Molecular Oncology, Beijing Institute for Cancer Research, School of Oncology, Peking University, Beijing, Hai-Dian District, 100080, China

**Keywords:** p42.3, Gastric cancer, Protein structure, Bayesian regulatory network

## Abstract

**Background:**

To analyze the p42.3 gene expression in gastric cancer (GC) cell, find the relationship between protein structure and function, establish the regulatory network of p42.3 protein molecule and then to obtain the optimal regulatory pathway.

**Methods:**

The expression of p42.3 gene was analyzed by RT-PCR, Western Blot and other biotechnologies. The relationship between the spatial conformation of p42.3 protein molecule and its function was analyzed using bioinformatics, MATLAB and related knowledge about protein structure and function. Furthermore, based on similarity algorithm of spatial layered spherical coordinate, we compared p42.3 molecule with several similar structured proteins which are known for the function, screened the characteristic nodes related to tumorigenesis and development, and established the multi variable relational model between p42.3 protein expression, cell cycle regulation and biological characteristics in the level of molecular regulatory networks. Finally, the optimal regulatory network was found by using Bayesian network.

**Results:**

(1) The expression amount of p42.3 in G1 and M phase was higher than that in S and G2 phase; (2) The space coordinate systems of different structural domains of p42.3 protein were established in Matlab7.0 software; (3) The optimal pathway of p42.3 gene in protein regulatory network in gastric cancer is Ras protein, Raf-1 protein, MEK, MAPK kinase, MAPK, tubulin, spindle protein, centromere protein and tumor.

**Conclusion:**

It is of vital significance for mechanism research to find out the action pathway of p42.3 in protein regulatory network, since p42.3 protein plays an important role in the generation and development of GC.

## Introduction

Gastric cancer (GC) is one of the most common malignant tumors in China and also in the world. Data showed that the new increasing GC patients are more than one million annually, with China accounts for 42%. About 0.8 million people dead of GC and 44% of them are in China. As one of the high GC incidence rate and death rate countries, the morbidity and mortality of China are more than twice of the world average level [1]. The tumorigenesis and development of GC is a complex issue involving genetic variation. Existing studies have demonstrated that genes, such as erbB-2, c-met, p53, cadherin, APC and RUNX3 gene, may be involved in the development and progression of GC [2,3].

The object of this research is the c9orf140 gene, which is located in the 9q34.3 site of the human chromosome, also a novel gene called p42.3 (DQ150361) which was cloned by synchronization, mRNA differential display and bioinformatics [4]. The full-length cDNA of p42.3 is approximately 4.0 kb, and the gene encodes a 389 amino acid protein that is estimated to have a molecular mass of 42.3 kDa. Further study found that its expression is cell cycle-dependent in GC cell lines. p42.3 protein expression peaks during the M phase of the cell cycle, then gradually declines after cell division; this indicates that p42.3 may be involved in cell cycle regulation. Furthermore, silencing of p42.3 by small interfering RNA results in the upregulation of CHK2 and the downregulation of cyclin B1, which are two key proteins involved in cell cycle regulation [5,6]. However, the mechanism of its action needs further exploration.

The biological function of protein is largely determined by its spatial structure. The research on the relationship between structure and function is the basis of protein function prediction and protein design. With the development of bioinformatics, mathematical method and computor technology are widely applied to protein structure prediction for less time-consuming and free from the constraints of experimental condition.

On the basis of establishing the model of protein structure domain spatial conformation and functional information, this study analyzed the regulatory function and mechanism of p42.3 protein in the malignant cell proliferation and tumor generation, it will provide theoretical basis for further experimental and clinical application.

## Materials

### Cell lines and tissue samples

GC cell line BGC823, MGC803, SGC7901, PAMC82, MKN45, SNU1, SNU5, SNU16, RF1, RF48, AGS and N87 are provided by Beijing Tumor Hospital.

### Reagents and main instruments

DMEM medium (Invitrogen, USA), Nocodazole (Sigma, USA), inverse transcription kit (Promega, USA), flow cytometry (Becton Dickinson, Flanklin Lakes, NJ, USA).

## Methods

### Cell culture and synchronization

GC cell lines were cultured in DMEM medium supplemented with 5% fetal bovine serum at 37°C in 5% CO2 incubator. The GC BGC823 cells were synchronized to G1, S, G2 and M phases by Nocodazole (mitotic inhibitor), then all synchronized cells were analyzed by FACS.

### Detection of the p42.3 mRNA expression level

The total RNA was extracted from each cell line and synthesized into cDNA through reverse transcription. Using β-actin as the control, PCR amplification was performed, and the product of which was conducted agarose gel electrophoresis detection. Moreover, the total RNA of BGC823 cells synchronized into each cell cycle was also extracted in order to detect the expression level in each phase by reverse transcription detection.

### Detection of the p42.3 protein expression

The law of p42.3 protein expression changes with cell cycle was analyzed by Western blotting.

### Analysis of protein structure

Protein folding was predicted by prediction tool Phyre in threading method. And Phyre2 was utilized to predict the tertiary protein structure and carry on the analysis for the secondary structure. The spatial conformation of protein was displayed by Swiss-Pdb Viewer 3.7 software.

### Protein similarity comparison

The similarity algorithm based on the spatial layered spherical coordinate: (1) A coordinate was built with the gravity center of the molecule as the coordinate origin. All atoms are transferred to this coordinate via the same transfer vector, and the conversion formula was 1.1, 1.2 and 1.3.

(2) Equidistant spherical shell layering was performed by the radius component in the spheric polar coordinate system. (3) The number of same C atom in each spatial layer is calculated separately. The summation of the number of C atom is computed and saved in the multi-dimensional vector *α*_*i*_ and *b*_*i*_. (4) The vector obtained in step 3 is substituted into function (1.4) in order to obtain the similarity value focused on the atom that composed by the weight. Then compute the weighted average; finally, the similarity value of protein molecule is carried out.

### Optimization of the regulatory network

Bayesian network is a kind of Directed Acyclic Graph (DAG) describing the joint probability distribution in the finite set of variables U = {X1, *X*2, …, Xn}. Bayesian network can be expressed by a pair of element B = (G, θ), where G is a DAG, the nodes in which are corresponding with the random variables X1, *X*2, …, Xn which can be the expression vector of gene in the gene expression data, while θ represents the conditional probability of each vector. DAG shows the following conditional independence relation, that is, Markove hypothesis: each variable Xi is conditionally independent of its non-descendants given its parent node in G. Based on the conditional independence property, the only joint probability distribution in set U of Bayesian network G is as follows:

(1) $$P\left(X1,X2,\ldots ,\mathrm{Xn}\right)=\prod_{i=l}^{n}P\left(\mathrm{Xi}|\mathrm{Pa}\left(\mathrm{Xi}\right)\right)\;$$

Where, Pa (Xi) represents the parent node of Xi. In order to verify the above joint probability, all the conditional probabilities in formula 1.3 should be defined. The core of theBayesian network is that this condition-independent probability relationship is interpreted as causal relationship, representing the causal regulatory relation between genes [7]. In this study, we utilized Bayesian network to find the optimal p42.3 regulatory pathway.

## Results

### Detection results of mRNA expression

**Figure 1:**
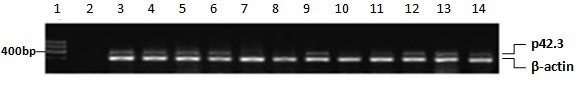
**p42.3 expression in GC cell lines.** 1: DNA marker 2: Negative control 3: AGS 4: MGC803 5: BGC823 6: SNU1 7: SNU5 8: SNU16 9: MKN45 10: RF1 11: RF48 12: N87 13: PAMC82 14: SGC7901.

**Figure 2:**
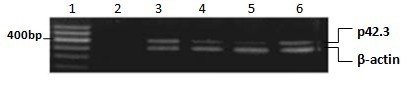
**p42.3 expression in each cell cycle.** 1: DNA marker 2: negative control 3:G1phase 4: S phase 5:G2 phase 6: M phase.

RT-PCR results indicated that p42.3 exists in most of the GC cells. In the selected 12 GC cell lines, p42.3 expression cannot be detected only in SNU5, SNU16 and RF1 cell lines (Figure 1); when BGC823 cells were synchronized to different cell cycles, results demonstrated that p42.3 expression in G1 and M phases was higher than that in S and G2 phases (Figure 2).

### Test results of p42.3 protein expression

**Figure 3:**
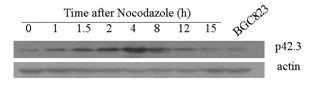
p42.3 protein expression analysis in different cell cycle.

BGC823 cells were synchronized to G1/S border zone by Nocodazole, and the cells gradually complete S phase, G2/M phase and G1phase with the withdrawing of drug. Within 24 hours after drug releasing, the cells were collected every two hours. P42.3 protein expression in the cell cycle was detected by Western blotting. The results indicated that p42.3 protein expression was cell-cycle dependent, barely expressing in S phase, highly expressing in the G2/M phase and early G1 phase and then declining. The p42.3 protein expression peaked in early G1 phase, then declined with G1 phase process (Figure 3).

### Analytical results of p42.3 protein structure

**Figure 4:**
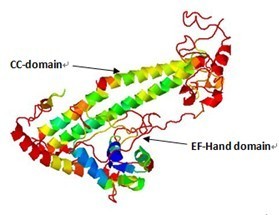
The spatial conformation of p42.3 molecule.

**Figure 5:**
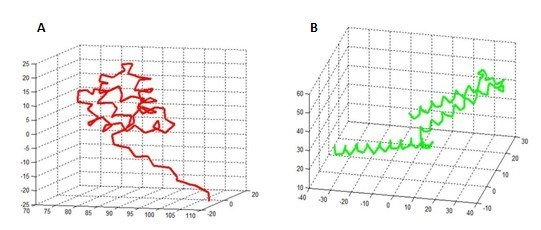
**Two characteristic structure domain analysis of p42.3 protein based on Matlab7.0.** α-C coordinate of EF-hand structure domain (B) α-C coordinate of CC-domain.

In the GenBank database, BLAST sequence homology search results indicated that p42.3 protein has no obvious homology with any amino acid sequence with known function. Therefore, we cannot use homology modelling to construct the three-dimensional structure of p42.3 molecule. The precise model cannot be built by ab initio prediction since the p42.3 protein amino acid sequence has 389 residues. Lack of homologous information, threading method was used to predict the folded structure of p42.3 molecules, which is, “threading” the amino acid sequence information of p42.3 molecules into the basic skeleton of the known protein and calculating the probability of each folding to predict the spatial conformation of p42.3 molecule structure domain. At first, multiple sequence alignment was performed by standard PSI-Blast method. Then, the structure comparison of the members in the family was conducted and output the sequence profile of this family. The 1D-3D sequence profile comparison between p42.3 molecule and template was performed, and the three-dimentional structure model of p42.3 molecule was established (Figure 4). Analysis of the structure data indicated that EF-hand structure domain existed in the N-end of the p42.3 protein. It has been reported that this conformation exists in the tumor-related calcium binding protein S100 family [8]. A CC domain, participating in the protein interaction and maybe under the regulation of phosphorylation, exists in the C-end of the p42.3 protein amino acid sequence. This three-dimentional conformation has high homology with the CC domain in the C-end of the APC molecule amino acid (95%). The spatial coordinate system of two structure domains of p42.3 protein established in Matlab7.0 was showed in Figure 5.

### Establishment of the regulatory network of p42.3 protein molecule functional domain group

The EF-Hand and CC-domain structure was screened, and the composed structure data set was shown in Tables 1 and 2. Using similarity algorithm based on spatial layered spherical coordinate and MATLAB software, we selected the following proteins which have high similarity with p42.3 protein and are related to tumorigenesis, namely, S100 family (including S100A1, S100A11, S100A2 and S100A4), small G protein, CIB (calmodulin-binding protein), OCK1 (Serine kinase), CENP-E (kinesin) and GCN4 protein were selected from the EF-Hand and CC-domain structure data set as the reference nodes for the nodes in tumorigenesis and development, which was sorted out as the protein regulatory network.

**Table 1:** Similarity comparison result in EF-hand data set

**Order**	** Symbol**	** Name**	**Similarity**	**Parameter**	** Remark**
1	CIB	Calcium and integrin binding protein	0.9742	4	calcium and integrin binding protein
2	SDF1	chemotatic factor	0.9263	4	Stromal cell-derived factor-1 Leukemia
3	S100A2	calcium binding protein	0.8994	4	
4	S100A11	calcium binding protein	0.8951	4	
5	RASEF	a kind of small G-protein	0.8817	4	RAS and EF-hand domain containing
6	MACF		0.7561	4	
7	KIAA		0.7374	4	family
8	S100A1	calcium binding protein	0.7230	4	
9	ATCN1		0.6475	4	
10	NLP		0.5094	4	
11	S100BCa	calcium binding protein	0.4900	4	
12	TNNC		0.4859	4	
13	MST3		0.4693	4	
14	GPD		0.4570	4	
15	S100A12	calcium binding protein	0.4561	4	
16	S100A4	calcium binding protein	0.4458	4	
17	KIAA-1837		0.4340	4	
18	PKD		0.4340	4	
19	REPS1		0.4109	4	Ral-binding protein
20	USP		0.4015	4	
21	FKBP		0.3227	4	

**Table 2:** Similarity comparison result in CC-domain data set

**Order**	** Symbol**	** Name**	**Similarity**	**Parameter**	** Remark**
1	CENP-E	kinesin	0. 8169	3	
2	GCN4		0.7611	3	monomer yeast transcriptional activator
3	ROCK1	Serine kinase	0.7394	3	Rho-associated,coiled-coil containing protein kinase 1
6	CENP-B		0.7297	3	
4	PAK1		0.6481	3	P21 activated kinase
7	GP41-HIV		0. 6010	3	
8	ATG12		0.4749	3	
5	ROCK2		0.4478	3	
9	CENP-A		0	0	

### Optimization of the p42.3 regulatory network

**Figure 6:**
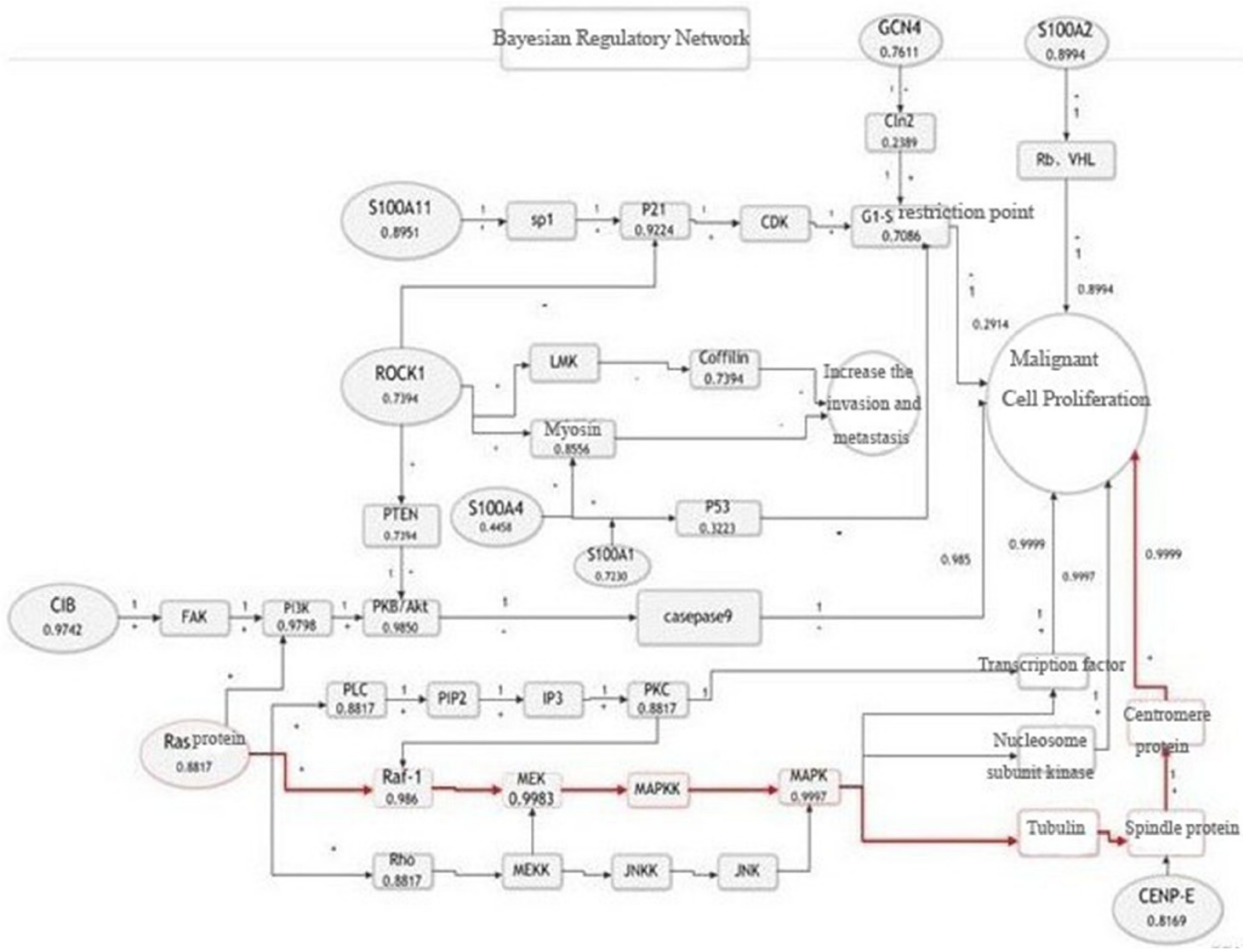
Bayesian regulatory network and optimal regulatory pathway of p42.3 protein.

The protein regulation network can show the action modes, pathway and mechanism of action of various kinds of factors; however, there is a certain distance to obtain the p42.3 protein regulation mode. Therefore, Bayesian network model can be applied to this model to optimize the model and find the optimal regulatory pathway. The optimized results and optimal regulatory pathway were shown in Figure 6.

As shown in Figure 6, we reversely seeked for the most possible reason leading to the final subevent – malignant cell proliferation, that is, the optimal regulatory pathway as the best prediction of the mechanism of action of p42.3 protein. After analysis, the optimal pathway marked by red was selected, that is, Ras protein–Raf-1 protein–MEK–MAPK kinase–MAPK–Tubulin–spindle protein– centromere protein–tumor showing in Figure 2, which is the most possible action pathway of p42.3.

## Discussion

P42.3 gene is highly reserved in mammalian. As an oncogene, p42.3 plays an important role in the transformation process from normal gastric epithelial cell to cancer cell. Cui Y et al. [9] found that MiR-29a can target at p42.3 gene. The p42.3 gene silencing can change the expression of two key genes -- CHK2 and cyclin B1, which would further inhibit cell proliferation and the advances of cell cycle. The above results verify that p42.3 plays an important role in cell cycle regulation.

Based on the theory that the protein’s function is determined by its spatial structure, we found molecules similar to p42.3 protein by bioinformatic software in related databank, and then predicted the spatial structure of p42.3 protein and analyzed the structure-function relation. Threading method was adopted to predict the spatial conformation of p42.3 gene for there is no homologous protein with p42.3 at present. Analysis of the structure data indicated that EF-hand structure domain existed in the N-end of the p42.3 protein. It has been reported that this conformation exists in the S100 family. A CC domain exists in the C-end, the three-dimentional conformation of which has high homology with the CC domain in the C-end of the APC molecule amino acid (95%). Study verified that the deactivation of APC gene plays an important role in the genesis and development of GC, which indicated that p42.3 protein may interfere related cell signal transduction pathway and biological function by influencing the active site of APC protein [10].

In protein molecule, easily distinguishable 3D structure is folded by two or more independent structure domains [11,12]. 3D structure exploring of protein molecule enables people to organize the rapidly growing set of thousands of known protein shapes, to identify new types of protein architecture, and to discover unexpected evolutionary relations [13]. The measurement of protein structure similarity influences the prediction and evaluation of the protein structure [14]. Hu min et al. [15] proposed a method for measuring protein structure similarity based on the molecular inner spatial density distribution, which the protein molecule space is performed concentric spherical shell division and got many shell structure space units with a certain radial depth, then the number of C atom in each spherical shell is counted. At last the spatial structure similarity of the two proteins is calculated by similarity calculation function. Zou BJ et al. [16] proposed a new method for measuring protein structure similarity based on spatial density characteristic, which is regarding spatial spherical polar coordinate as the presentation model. The model based on spherical polar coordinate can provide theoretical basis for protein classification and protein function prediction. We transfer the protein 3D structure information into distance sequence information which is then performed fast Fourier transform, and the spatial domain information of protein structure is transformed into frequency domain information. At last, the similarity of spatial domain information is determined by the similarity of frequency domain information [17].

The construction of gene regulatory network is based on the gene expression data with every gene as a random variable and the expression level in different conditions as its value. Moreover, gene regulatory network is sparsity [18], which means, one a few genes directly affect the transcription process. Having the characteristic of illustrating the probability dependence of variables in the form of possibility, Bayesian Network is very suitable for analyzing and predicting this kind of sparse network.

Based on the two characteristic structures (EF-hand structure domain and CC domain) of p42.3 protein, we collected a large amount of similar protein. The similar protein set was screened by protein structure similarity comparison method based on spherical polar coordinate. The constructed regulatory network was optimized by Bayesian Network in order to obtain the optimal regulatory pathway. The possible action way of p42.3 gene in tumorigenesis was explored, which provided powerful means for the further study on mechanism of action. Applying mathematics and computer technolgoy in protein structure prediction and function analysis is a new trend in the field of biomedical research.

## Abbreviation

GC: Gastric cancer.

## Competing interests

The authors declare that they have no competing interests.

## Authors’ contributions

JH Zhang and CL Lu drafted the manuscript. ZG Shang participated in the design of the study and performed the statistical analysis. R Xing conceived of the study, and participated in its design and coordination and helped to draft the manuscript. YY Lv and L Shi designed the study. All authors read and approved the final manuscript.
